# Past, Present, and Possible Future Human Infection with Influenza Virus A Subtype H7

**DOI:** 10.3201/eid1506.090072

**Published:** 2009-06

**Authors:** Jessica A. Belser, Carolyn B. Bridges, Jacqueline M. Katz, Terrence M. Tumpey

**Affiliations:** Centers for Disease Control and Prevention, Atlanta, Georgia, USA (J.A. Belser, C.B. Bridges, J.M. Katz, T.M. Tumpey); Mount Sinai School of Medicine, New York, New York, USA (J.A. Belser)

**Keywords:** Influenza, pandemic, bioterrorism and preparedness, conjunctivitis, pathogenesis, subtype, viruses, perspective

## Abstract

Influenza A subtype H7 viruses have resulted in >100 cases of human infection since 2002 in the Netherlands, Italy, Canada, the United States, and the United Kingdom. Clinical illness from subtype H7 infection ranges from conjunctivitis to mild upper respiratory illness to pneumonia. Although subtype H7 infections have resulted in a smaller proportion of hospitalizations and deaths in humans than those caused by subtype H5N1, some subtype H7 strains appear more adapted for human infection on the basis of their virus-binding properties and illness rates among exposed persons. Moreover, increased isolation of subtype H7 influenza viruses from poultry and the ability of this subtype to cause severe human disease underscore the need for continued surveillance and characterization of these viruses. We review the history of human infection caused by subtype H7. In addition, we discuss recently identified molecular correlates of subtype H7 virus pathogenesis and assess current measures to prevent future subtype H7 virus infection.

## Abstract 2

These viruses have resulted in >100 cases of human infection since 2002, and their pandemic potential should not be underestimated.

Influenza A viruses belong to the family *Orthomyxoviridae* and possess 8 negative-sense RNA segments encoding 11 known proteins. Of these, the 2 viral surface glycoproteins, hemagglutinin (HA) and neuraminidase (NA), form the basis of multiple serologically distinct virus subtypes. Currently, 16 HA and 9 NA subtypes have been identified in wild water birds, the natural host for all influenza A viruses and the reservoir from which viruses emerge to infect domestic poultry and occasionally mammals. Most influenza viruses that infect wild or domestic birds cause no or limited illnesses and deaths and are characterized as being low pathogenicity avian influenza (LPAI) viruses. However, viruses within the H5 and H7 subtypes have the capacity to acquire genetic properties that confer high virulence and a high proportion of deaths in chickens and other fowl after their introduction into domestic poultry; these viruses are characterized as highly pathogenic avian influenza (HPAI) viruses according to the intravenous pathogenicity index method described by the World Organization for Animal Health (*1*). LPAI viruses (H9N2) are also prevalent in poultry in many countries and are considered to have pandemic potential (*2*). Domesticated birds may serve as important intermediate hosts for the transmission of wild bird influenza viruses to humans, as may swine, as evidenced by recent human infections with swine influenza virus A (H1N1) on multiple continents. In April 2009, the World Health Organization (WHO) reported human illness caused by a new strain of swine influenza virus subtype H1N1; infections were soon confirmed in 7 countries. As of April 28, 2009, Mexico had reported the highest number of subtype H1N1 cases, with 26 confirmed human cases of infection and 7 deaths..

If an influenza virus with an HA against which the human population had little or no immunity crossed the species barrier and was efficiently transmitted among humans, a pandemic could result. Three pandemics occurred in the 20th century: in 1918 (H1N1), 1957 (H2N2), and 1968 (H3N2). However, none of these pandemic strains possessed the HA cleavage site mutation characteristic of HPAI viruses (*3*). Thus, the HPAI phenotype is not required for an influenza virus to cause a pandemic. Three HA subtypes, H1–H3, subsequently established stable lineages in humans; 2 subtypes, H1N1 and H3N2, cause seasonal epidemics today, which result in ≈36,000 deaths in the United States annually (*4*). Although the severity of a pandemic virus cannot be known in advance, attack rates could reach 25%–35%. The resulting surge in the number of persons requiring medical or hospital treatment would undoubtedly overwhelm the healthcare system.

Within the past decade, HPAI and LPAI viruses have been found to be associated with human infection, primarily as a result of direct transmission from poultry to humans (*2*,*5*,*6*). However, none of these viruses have yet acquired the ability to be transmitted efficiently among humans. LPAI viruses of the H7 and H9 subtypes have caused mild respiratory or conjunctival infections in humans. However, some HPAI subtype H5 and H7 viruses, which cause a high proportion of deaths in experimentally infected chickens, have been associated with severe human disease and death (*5*,*6*). Due to an unprecedented geographic expansion of subtype H5N1 viruses since 2003 and continued sporadic human subtype H5N1 infections, much emphasis has been placed on the potential pandemic threat posed by subtype H5N1 viruses. In contrast, subtype H7 infection in humans has not been as extensively studied. In this perspective, we will discuss the epidemiology of subtype H7 in humans, current research that explores the pandemic potential of these viruses, and ongoing measures to prevent future human infection.

## Prevalence of Subtype H7 Influenza Viruses in Poultry and Risk for Human Infection

Subtype H7 influenza viruses, like avian influenza viruses of all subtypes, fall into 2 geographically distinct genetic lineages, North American or Eurasian (*7*). Viruses within both lineages have been associated with human infection (Table). In recent years, poultry outbreaks caused by HPAI and LPAI viruses of the H7N1, H7N2, H7N3, H7N4, and H7N7 subtypes have resulted in the culling of >75 million birds (*18*). Notably, the geographic diversity of countries affected by the H7 subtype in poultry, which includes Pakistan, Australia, Ireland, Italy, Canada, Germany, Chile, the Netherlands, and the United States, readily demonstrates the global public health risk posed by viruses within this subtype (*18*).

**Table:** Cases of human subtype H7 influenza A virus infection since 1996*

Location	Year	Subtype	IVPI	No. human infections	Symptoms	References
UK (England)	1996	H7N7	LPAI	1	Conjunctivitis	(*8*,*9*)
USA (Virginia)	2002	H7N2	LPAI	1†	Respiratory	(*10*)
USA (New York)	2003	H7N2	LPAI	1	Respiratory	(*11*)
Italy	2002–03	H7N3	LPAI	7†	Conjunctivitis, respiratory	(*12*)
The Netherlands	2003	H7N7	HPAI	89	Conjunctivitis, respiratory	(*6*,*13*)
Canada (British Columbia)	2004	H7N3	LPAI/HPAI	2	Conjunctivitis, respiratory	(*14*,*15*)
UK (Norfolk)	2006	H7N3	LPAI	1	Conjunctivitis	(*16*)
UK (Wales)	2007	H7N2	LPAI	4	Conjunctivitis, respiratory	(*17*)

*IVPI, intravenous pathogenicity index (*1*); LPAI, low pathogenicity avian influenza; HPAI, highly pathogenic avian influenza. †Serologic evidence only.

Before 2003, reports of subtype H7 infection in humans were rare and primarily resulted from laboratory or occupational exposure. One exception was the first documented isolation of a fowl plague-like virus (FPV; HPAI viruses of the H7N7 subtype) from a human, which occurred in the United States in 1959, from the blood of a man with clinically diagnosed infectious hepatitis (*19*,*20*). In 1977, a laboratory technician became infected through accidentally splashing allantoic fluid containing FPV on her face, which resulted in conjunctival symptoms (*21*). During the winter of 1979–80, a virus antigenically similar to A/fowl plague/Dutch/27 (H7N7) caused the deaths of ≈500 seals on the New England coast**.** Subsequent study of the prototype virus A/seal/Massachusetts/1/80 (H7N7) resulted in the infection of a laboratory worker when an experimentally infected seal sneezed into the face and the right eye of the worker (*22*,*23*). Four persons who conducted necropsies of infected seals also contracted conjunctivitis within 2 days of known ocular exposure; although the virus was not isolated from the 4 field workers, clinical signs and duration of illness were consistent with subtype H7N7 virus infection (*22*).

The first reported case of direct transmission of a subtype H7 virus from an avian to a human host occurred in 1996, when conjunctivitis developed in a woman who kept pet ducks 1 day after she experienced a possible eye abrasion while cleaning her duck house (*8*,*9*). A conjunctival swab from this patient was found to be positive for an influenza virus A (H7N7), A/England/268/96, which was determined to be wholly avian in origin by sequence analysis (*9*). However, a rise in serum hemagglutination inhibition (HI) titer to virus postexposure was not detected in any of these early human infections. It is not known whether the absence of HI antibody detected in serum specimens from these infected persons was due to an actual lack of induction of serum antibodies after infection with these H7 subtypes or whether the relative insensitivity of the avian erythrocyte-based HI assay used at that time contributed to these findings. Nevertheless, these initial events clearly confirmed the ability for interspecies transmission of subtype H7 viruses to humans.

## Recent Human Infections with Subtype H7 Influenza A Viruses

In contrast to these isolated instances of human infection with subtype H7 viruses, numerous outbreaks of LPAI and HPAI viruses of this type among poultry since 2000 have resulted in increased numbers of human exposure and infection (Table). This increase in detection may be a result of a combination of several factors: more human infections, improved PCR diagnostic testing, heightened awareness of the risk for avian influenza in humans caused by subtype H5N1, and increased surveillance and testing of humans exposed to avian influenza. The largest outbreak of subtype H7 infections in humans to date occurred in the spring of 2003, when an HPAI (H7N7) virus was detected in commercial poultry farms in the Netherlands and necessitated the culling of >30 million birds (*6*,*13*). All internal genes of this virus were of avian origin and were found to be related to low pathogenicity viruses detected during surveillance of ducks in the region in 2000 (*13*). Eighty-six persons involved in the culling operation and 3 of their family members who had not been in contact with infected poultry had virologically confirmed subtype H7 illness, which suggests that limited human-to-human transmission of the avian virus also had occurred (*6*). Among these persons, 78 had conjunctivitis, 5 had conjunctivitis and respiratory symptoms, 2 had respiratory symptoms only, and 1 died (*6*), a veterinarian who had visited several infected farms and in whom an acute respiratory distress syndrome and pneumonia developed. The virus isolated from a postmortem lung specimen of the patient with the fatal case, A/NL/219/2003, differed by 14 aa residues across 5 gene segments from a virus isolated from a chicken on the index farm, A/ck/NL/1/2003 (*6*). Serologic studies have provided further evidence of human infection during this outbreak (*24*). The number of human illnesses in this outbreak is in stark contrast to outbreaks of subtype H5N1 infection; most human cases of influenza virus A (H5N1) have occurred as isolated cases or small clusters of <3 cases with a maximum of 8 persons clinically ill (*5*).

In addition to the HPAI (H7N7) outbreak in the Netherlands, LPAI (H7N3) viruses caused outbreaks in poultry in northern Italy during 2002–03. Retrospective serologic analysis of workers involved in the outbreak response identified 7 of 185 persons who had close direct physical contact with poultry and were seropositive by microneutralization assay and Western blot analysis for subtype H7 influenza (*12*). One of these persons reported conjunctival symptoms during the outbreak. However, seroreactivity was not detected in workers involved in the earlier outbreak responses to LPAI and HPAI viruses (H7N1) that caused multiple poultry outbreaks in Italy from 1999–2001, which suggests either a different level of human exposure to subtype H7N1 viruses or differing abilities of subtype H7 viruses to transmit to humans (*12*).

HPAI and LPAI subtype H7 viruses have also caused poultry outbreaks and economic loss in the Americas. LPAI viruses (H7N2) have circulated in the northeastern United States live bird markets for over a decade and were the cause of a devastating outbreak predominantly on domestic turkey farms in 2002. One of 80 tested workers involved in the culling operations during this outbreak reported a temporally related respiratory illness and exhibited serum-neutralizing antibody responses consistent with a subtype H7N2 virus infection, providing the first evidence of possible human infection with a North American lineage LPAI virus (H7N2) (*10*). One year later, an immunocompromised New York resident with a fever and cough sought treatment at a hospital, and a subtype H7N2 virus, A/NY/107/2003 (NY/107), was subsequently isolated from a respiratory specimen (*11*). The HA gene of NY/107 virus exhibits 98% aa sequence identity with a representative virus from the 2002 outbreak in Virginia, A/tky/VA/4529/02 (*25*). The person recovered from the respiratory illness and demonstrated seroconversion to subtype H7N2 (NY/107) virus, but the source of his initial exposure to the avian virus remains unknown. LPAI virus (H7N2) was isolated from 133 of 4,675 poultry specimens from New York, and 1 of 3,406 specimens from New Jersey in early 2006, but this subtype has not been detected among domestic poultry in the United States since March 2006 (*26*).

HPAI subtype H7 viruses again caused human disease in North America, as observed in Spring 2004 during an outbreak of subtype H7N3 infection in poultry in British Columbia, Canada. The initial virus was an LPAI virus that subsequently became HPAI by acquisition of a 7-aa M1 gene sequence insertion at the HA cleavage site through a nonhomologous recombination event (*14*). Among workers associated with the outbreak response, 57 suspected human cases of subtype H7N3 infection were reported due to conjunctival or influenza-like illness symptoms (*15*). In 2 of these persons, who were involved in the culling of infected birds, conjunctivitis developed after direct ocular exposure to infected poultry after a breach in eye protection. Influenza virus A (H7N3) was isolated from a nasal specimen from 1 person with conjunctivitis and coryza, A/Canada/444/2004 (Can/444), and another from a conjunctival specimen from the other person who exhibited conjunctivitis and headache, A/Canada/504/2004 (Can/504); both persons recovered fully (*15*). Although both human isolates contained the 7-aa M1 gene sequence insertion, an intravenous pathogenicity index test determined that Can/504 was HPAI, whereas Can/444 was not (*14*). Notably, the emergence of HPAI from LPAI viruses by nonhomologous recombination has been reported with both North American and Eurasian lineage subtype H7 viruses, but not viruses within the H5 subtype (*14*,*27*).

Most recently, multiple H7 viruses have resulted in cases of human infection in the United Kingdom. In 2006, an LPAI virus (H7N3) first detected in a poultry flock in eastern England was isolated from a poultry worker with conjunctivitis (*16*). Four additional persons associated with the outbreak later presented with conjunctivitis or influenza-like illness, but all symptomatic persons were PCR negative for influenza (*16*). In 2007, poultry infected with LPAI (H7N2) were sold from a small market in the United Kingdom and resulted in 4 persons with confirmed cases of H7 human infection, 3 of whom were hospitalized for 3–7 days, and 19 additional symptomatic persons for whom PCR results were negative (*17*). Those exposed to the virus reported both conjunctivitis and influenza-like illness; one of the hospitalized patients had neurologic and gastrointestinal symptoms, but not respiratory disease (*28*). The increased frequency of human infection with H7 viruses in recent years, coupled with the continued detection of H7 influenza viruses in poultry in both Europe and North America, suggests that future human infections with viruses within this subtype are likely to occur.

Surprisingly, seroconversion for neutralizing antibody has rarely been observed among persons with virologically confirmed subtype H7 infection. For example, neutralizing antibody responses were not detected in persons confirmed to be infected with the HPAI virus (H7N7) in 2003 (*24*). Likewise, neutralizing antibody titers were not detected in convalescent-phase serum from any person exposed to infected birds during the 2004 subtype H7N3 outbreak in Canada, including those with confirmed cases with positive virus isolation (*15*,*29*). In contrast, infection with LPAI (H7N2) that resulted in respiratory illness in the United States did induce a detectable serum-neutralizing antibody response (*10*). Low antibody levels also were detected in a person who was infected with an LPAI virus (H7N3) (*30*). However, the optimal methods of detecting antibody and criteria for seropositivity to H7 virus in humans remain unclear; current criteria used are those established and adapted by the WHO for H5N1 subtype human infection and extrapolated for the H7 subtype. In addition to evaluating potential avian subtype-specific differences in the detection of neutralizing antibodies, further study is needed to ascertain whether conjunctival avian virus infection routinely leads to detectable serum antibodies. Sensitive and specific methods of detecting mucosal antibody to influenza virus in ocular specimens are also needed.

## Properties of Subtype H7 Influenza Viruses

Because of the sustained frequency of epornitics caused by influenza virus subtype H5N1 that have resulted in human infections during the past 5 years, viruses within this subtype are rightly considered a major pandemic threat. However, subtype H7 influenza viruses share many properties with viruses within the H5 subtype, and H7 outbreaks involving large numbers of infected persons have been documented. Thus, the pandemic potential of subtype H7 viruses should not be underestimated because viruses within this subtype have caused severe human infection and death, with limited human-to-human transmission (Figure) (*5*,*13*). Interestingly, although subtype H5N1 infection most frequently manifests as severe respiratory disease, human infection with subtype H7 viruses predominantly result in conjunctivital symptoms with occasional and generally mild respiratory illness. Despite the overall differences in human disease manifestations and severity, subtype H7 viruses can replicate efficiently in the respiratory tract of experimentally infected animals without the need for prior adaptation, and have the capacity to spread systemically, including to the central nervous system, in mammalian models (*31*,*32*).

**Figure:**
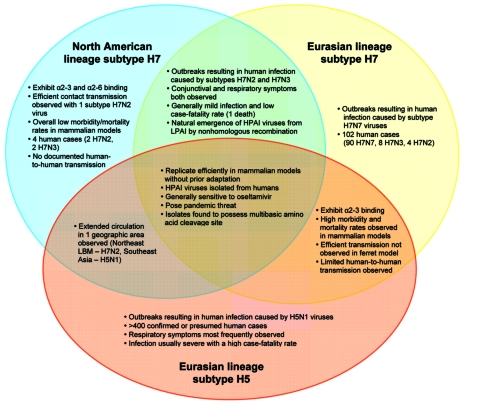
Public health impact of influenza virus A subtypes H7 and H5. HPAI, highly pathogenic avian influenza; LPAI, low pathogenicity avian influenza; LBM, live bird market.

North American lineage subtype H7 viruses, despite exhibiting reduced virulence in mammalian models as compared with subtype H7 viruses from the Eurasian lineage, nonetheless possess multiple features that underscore the public health threat posed by these viruses. This is especially apparent for the North American lineage LPAI viruses (H7N2), which circulated in the live bird markets of the northeastern United States for over a decade. These viruses possess a 24-nt deletion found in the HA and a 51-nt stalk deletion in the NA, which distinguishes them from other subtype H7 viruses found in domestic poultry in North America. Since these viruses were introduced in 1994, the HA cleavage site of circulating viruses acquired additional basic amino acids, a known correlate of pathogenicity for avian influenza viruses (*33*).

Recent work has also identified that contemporary North American lineage subtype H7 viruses, isolated in 2002–03, are partially adapted to recognize α2–6 linked sialic acids, which are the receptors preferred by human influenza viruses and found in the human upper respiratory tract (*34*). A critical determinant for viral transmission among humans believed to be the binding between the virus and sialic acid receptors located on cells in the upper airway. Therefore, if North American lineage subtype H7 viruses adapt further to enhance their ability to bind solely to α2–6 linked sialic acid receptors, these avian influenza viruses could have the potential to spread more efficiently from birds to humans and among humans. Although human-to-human transmission has not been documented among North American lineage subtype H7 influenza viruses, the discovery of an LPAI virus (H7N2) isolated from a human in 2003 that was transmissible by direct contact in ferrets identifies the potential of viruses within this lineage to acquire this property (*34*). In contrast, most avian influenza viruses tested in this manner fail to transmit. Human influenza viruses are thought to be transmitted primarily by respiratory droplets expelled during coughing or sneezing; no avian viruses of subtype H5 or H7 have yet demonstrated the ability to spread through respiratory droplets in the ferret transmission model.

In comparison with the generally mild infections observed with either HPAI or LPAI North American lineage subtype H7 viruses, HPAI (H7N7) European lineage viruses isolated from humans resemble subtype H5N1 viruses in their capacity for high virulence in mammalian models (*31*,*32*). Eurasian lineage subtype H5N1 viruses have been found to be more virulent in the mouse model than non-Eurasian lineage subtype H5N1 viruses; however, no molecular determinants have been associated with the hypothesis that Eurasian lineage avian influenza viruses are more capable of infecting mammals (*35*). Nevertheless, selected subtype H7 viruses within the Eurasian lineage possess molecular features (such as the E627K PB2 substitution) most frequently found in highly pathogenic subtype H5N1 viruses in poultry and, additionally, resemble highly pathogenic subtype H5N1 viruses with regard to the preservation of an avian receptor-binding preference and a general inability to transmit efficiently in the ferret model (*34*,*36*).

Despite >400 confirmed human cases of subtype H5 infection since 1997, all infections have resulted from viruses possessing the N1 NA subtype (*5*). In contrast, subtype H7 viruses with multiple NA subtypes have successfully transmitted from birds to humans, suggesting that multiple NA subtypes are compatible with the subtype H7 HA. Although subtype H5N1 viruses associated with disease in humans have predominantly been HPAI viruses from the Eurasian lineage, both lineages of subtype H7 viruses have been associated with disease in humans. The great diversity of subtype H7 viruses associated with disease in humans supports the need for active surveillance for illness among persons exposed to subtype H7 viruses, including farm workers, cullers, and the families of these workers, as well as healthcare providers who care for ill persons involved in subtype H7 outbreaks. The occurrence of conjunctival symptoms after infection with subtype H7 viruses, a clinical sign of illness not frequently associated with infection with other virus subtypes, further demonstrates the complexity of this virus subtype; research investigating the ocular tropism of selected influenza viruses is needed to better understand and protect humans from this possible route of virus entry.

## Preventing Subtype H7 Virus Infection in Humans

Although effective vaccines offer the best protection against avian influenza viruses, technical limitations currently prevent the rapid generation and availability of a strain-specific vaccine against an emerging pandemic virus. The emergence of multiple antigenically distinct virus clades, resulting in a need for clade-specific vaccine candidates, has posed a substantial challenge for the design of subtype H5N1 virus vaccines (*5*). The generation of subtype H7 vaccine candidates faces similar challenges because antigenically distinct subtype H7 lineages have resulted in human disease, and the isolation of North American lineage subtypes H7N2, H7N3, and Eurasian lineage H7N7 and H7N3 viruses from humans in recent years identifies multiple distinct H7 subtypes that may warrant the development of appropriate vaccine candidates. Vaccination of poultry has been successful in controlling of subtype H7 influenza (*18*); vaccines for human use against both lineages of H7 influenza are under development and have been evaluated in preclinical studies (*25*,*32*,*37*).

Antiviral strategies that are effective against influenza viruses of multiple subtypes will be an important first line of defense in the event of a pandemic. Unfortunately, the emergence of antiviral-resistant subtype H5 and H7 influenza viruses has been documented. Viruses from the 2003 Netherlands outbreak were found to be sensitive to the NA inhibitors oseltamivir and zanamivir in vitro but resistant to the M2 ion-channel blocker amantadine both in vitro and in a mouse model (*13*,*38*). Amantadine-resistant variants have also been observed among subtype H7 viruses within the North American lineage (*39*). Together with the detection of subtype H5N1 viruses with reduced susceptibility to antiviral agents (*5*), these findings underscore the importance of surveillance for resistant viruses of avian influenza virus of multiple subtypes as well as the generation of novel antiviral strategies to combat influenza viruses of an unknown subtype.

In addition to pharmacologic interventions, the correct use of personal protective equipment during possible virus exposure should be emphasized. The frequency of conjunctival symptoms after subtype H7 virus exposure underscores the importance of protecting the ocular surface from possible abrasion and virus entry; eye protection is recommended for all persons during possible exposure to avian influenza viruses (*40*). Given the potential for human infection, active monitoring for illness and for adherence to appropriate use of personal protective equipment among all persons potentially exposed to subtype H7 viruses during outbreaks in poultry should be conducted, and testing should be readily available should illnesses occur.

Subtype H5N1 viruses are now endemic in countries in Asia and Africa, and subtype H7 viruses continue to circulate across Europe and North America, as demonstrated by the detection of subtype H7 influenza viruses in chickens in Arkansas and the United Kingdom, and swans in Rhode Island, during the summer of 2008. Future human infection with viruses of both subtypes will likely continue to occur. It is clear that the study of avian influenza viruses (H5N1) has greatly improved our understanding of avian viruses. Applying this knowledge toward the assessment of other HPAI and LPAI viruses with pandemic potential, such as those within the H7 subtype, will further improve our ability to respond to and reduce the severity of future pandemics, regardless of virus subtype.
